# Supplementary figures and images for: Rats that learn to vocalize for food reward emit longer and louder appetitive calls and fewer short aversive calls
Source: PLoS One. 2024 Feb 9;19(2):e0297174. doi: 10.1371/journal.pone.0297174 (PMC10857575; doi:10.1371/journal.pone.0297174)

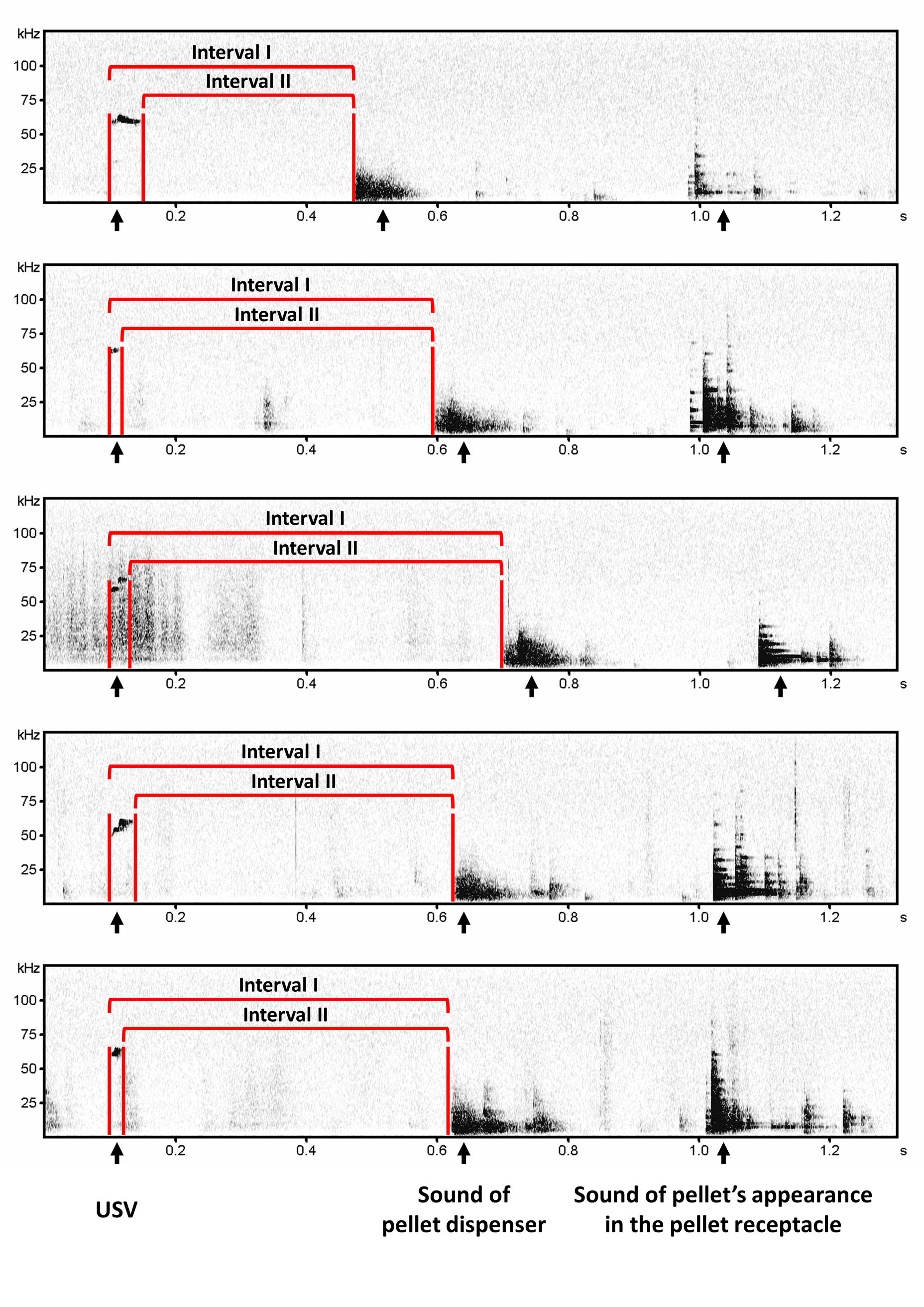

Supplement: S1 Fig — Interval I–between the start of the rewarded USV and the sound of the pellet dispenser; Interval II–between the end of the rewarded USV and the sound of the pellet dispenser. First arrows point the USV, second arrows point the sounds of pellet dispenser and third arrows point the sounds of pellet’s appearance in the pellet receptacle; see also Experimental schemes: USV-training in Experimental Procedures. (TIF) [file pone.0297174.s001.tif]

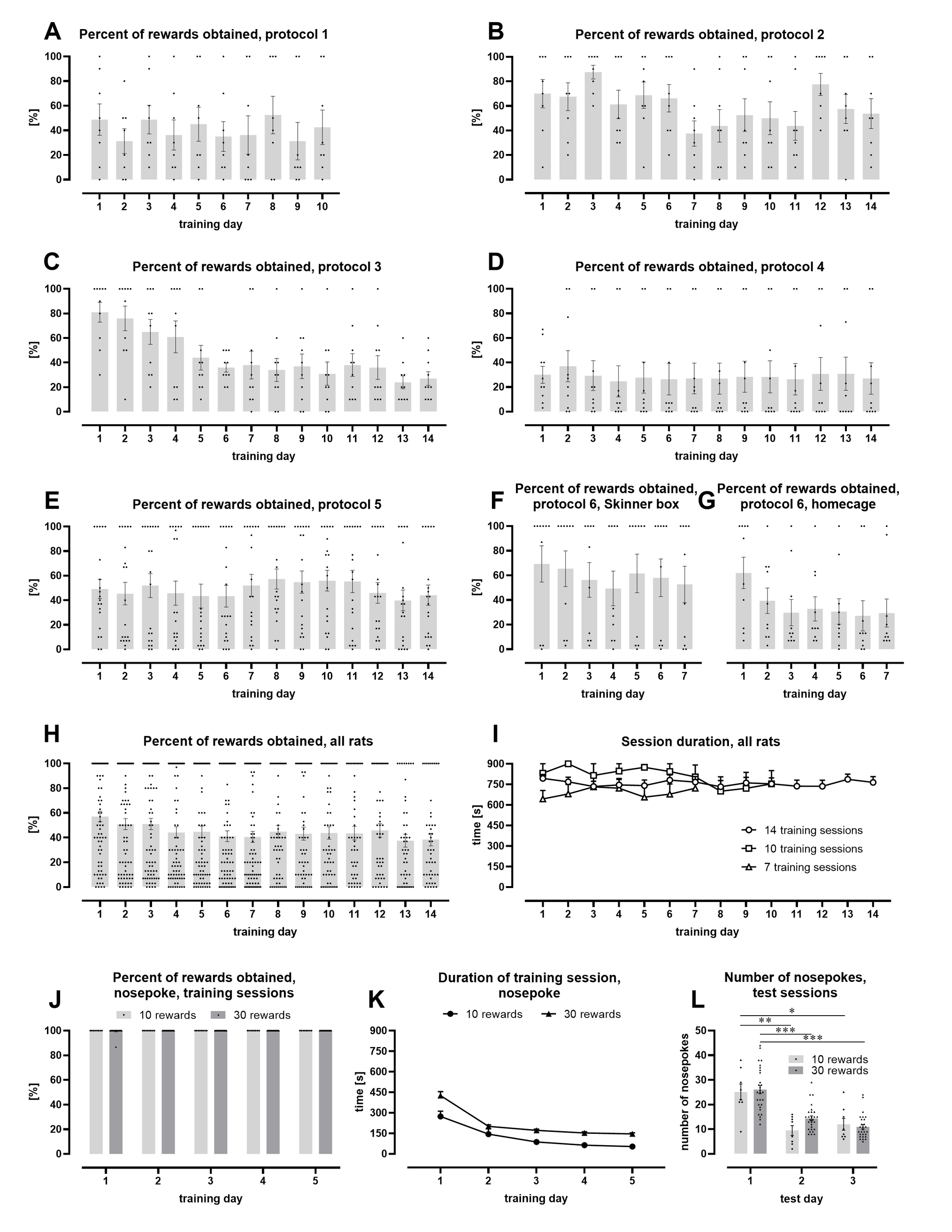

Supplement: S2 Fig — Changes in the percentage of rewards rats obtained and the duration of training sessions in operant-conditioning protocols 1–6 with vocalization emissions (A-I) or nosepokes (J-L) as rewarded responses. Rats could obtain a maximum of 10 or 30 rewards in one training session. A training session was terminated when a rat obtained the maximum number of rewards or the trial time exceeded 15 min. A. Protocol 1 (see also Fig 1 and Table 1 for protocols’ description): Food-restricted (95% initial body weight) rat was trained for 10 sessions, with a maximum of 10 rewards. B. Protocol 2: Conditions from Protocol 1 were modified by increasing food deprivation to 90% and extending training to 14 sessions. C. Protocol 3: Conditions from Protocol 2 were modified by adding 2 vocalization-eliciting stimuli: bedding from a cagemate and USV playback. D. Protocol 4: Conditions from Protocol 3 were modified by increasing the maximum number of rewards to 30. E. Protocol 5: Conditions from Protocol 4 were modified by performing training sessions in the dark phase instead of the light phase. F. Protocol 6: Conditions from Protocol 5 were modified by using habituation to the experimental cage (4 sessions before training) and reducing the training to 7 sessions. G. Protocol 6: Conditions were the same as in F, with habituation and training in a cage identical to the home cage. In all protocols, there was no overall increase in the number of rewards obtained (A, B, D, E, F), rather a decrease was observed (C: p < 0.0001, Friedman, p = 0.0020, Wilcoxon; G: p = 0.0143, Friedman, p = 0.0078, Wilcoxon). H. Percent of rewards obtained during training from all protocols (1–6, A-G) pooled together; there was a decrease in the number of rewards (days 1–7: p < 0.0089, Friedman; p = 0.0015, Wilcoxon; days 1–14: p < 0.0001, Friedman; p = 0.0022, Wilcoxon, S2b Table). I. Duration of training sessions from all protocols (1–6, A-G) showed no change in time (days 1–7: p = 0.7292, Friedman; p = 0.2324, Wil [file pone.0297174.s002.tif]

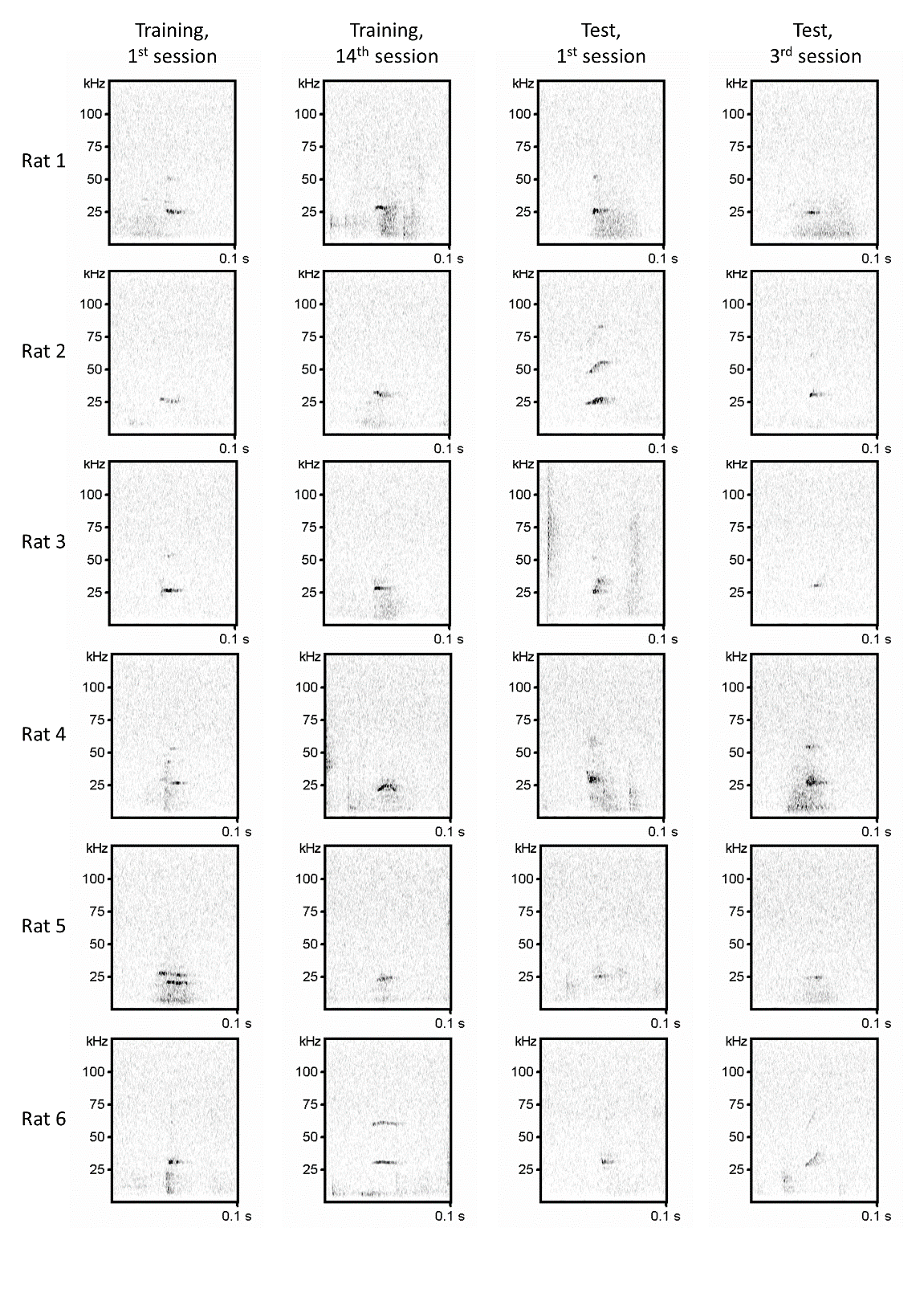

Supplement: S3 Fig — Emitted by non-learning rats (Nos. 1–3) and potentially learning rats (Nos. 4–6) during the first and last training sessions (first two columns) as well as the first and last test sessions (latter two columns). (TIF) [file pone.0297174.s003.tif]
