# Supplementary material for: Rats that learn to vocalize for food reward emit longer and louder appetitive calls and fewer short aversive calls
Source: PLoS One. 2024 Feb 9;19(2):e0297174. doi: 10.1371/journal.pone.0297174 (PMC10857575; doi:10.1371/journal.pone.0297174)
Supplement: S1 Table — (PDF) [file pone.0297174.s004.pdf]

S1 Table. Percentage of rewards obtained by each rat during each training session. Next to the rat numbers, protocol numbers are given in brackets. Days when a rat obtained 100% of the rewards are marked in bold.

**PL rats**														
group	**PL-PROG**													
rat	training day													
	**1**	**2**	**3**	**4**	**5**	**6**	**7**	**8**	**9**	**10**	**11**	**12**	**13**	**14**
1 (2)	10.0	50.0	60.0	40.0	10.0	20.0	20.0	40.0	40.0	10.0	30.0	**100.0**	**100.0**	**100.0**
2 (2)	80.0	70.0	80.0	40.0	**100.0**	**100.0**	90.0	90.0	90.0	80.0	90.0	**100.0**	**100.0**	**100.0**
3 (4)	43.3	**100.0**	**100.0**	**100.0**	**100.0**	**100.0**	**100.0**	**100.0**	**100.0**	**100.0**	**100.0**	**100.0**	**100.0**	**100.0**
4 (4)	66.7	**100.0**	**100.0**	**100.0**	**100.0**	**100.0**	**100.0**	**100.0**	**100.0**	**100.0**	**100.0**	**100.0**	**100.0**	**100.0**
5 (5)	36.7	83.3	**100.0**	**100.0**	**100.0**	**100.0**	**100.0**	**100.0**	**100.0**	**100.0**	**100.0**	**100.0**	**100.0**	**100.0**
6 (5)	16.7	3.3	3.3	0.0	13.3	20.0	**100.0**	**100.0**	**100.0**	**100.0**	**100.0**	**100.0**	**100.0**	**100.0**
7 (1)	90.0	40.0	50.0	70.0	60.0	**100.0**	**100.0**	**100.0**	**100.0**	**100.0**				
8 (1)	50.0	50.0	90.0	**100.0**	**100.0**	70.0	**100.0**	**100.0**	**100.0**	**100.0**				
9 (6)	**100.0**	**100.0**	50.0	33.3	**100.0**	**100.0**	**100.0**							
10 (6)	**100.0**	**100.0**	83.3	26.7	**100.0**	**100.0**	**100.0**							
group	**PL-MAX**													
rat	training day													
	**1**	**2**	**3**	**4**	**5**	**6**	**7**	**8**	**9**	**10**	**11**	**12**	**13**	**14**
11 (5)	**100.0**	**100.0**	**100.0**	**100.0**	**100.0**	**100.0**	**100.0**	**100.0**	**100.0**	**100.0**	**100.0**	**100.0**	**100.0**	**100.0**
12 (5)	**100.0**	**100.0**	**100.0**	**100.0**	**100.0**	**100.0**	**100.0**	**100.0**	**100.0**	**100.0**	**100.0**	**100.0**	**100.0**	**100.0**
13 (6)	**100.0**	**100.0**	**100.0**	**100.0**	**100.0**	**100.0**	**100.0**							
14 (6)	**100.0**	**100.0**	**100.0**	**100.0**	**100.0**	**100.0**	**100.0**							
15 (6)	**100.0**	**100.0**	**100.0**	**100.0**	**100.0**	**100.0**	**100.0**							

**NL rats**														
group	**NL-0**													
rat	training day													
	**1**	**2**	**3**	**4**	**5**	**6**	**7**	**8**	**9**	**10**	**11**	**12**	**13**	**14**
1 (2)	40.0	70.0	90.0	30.0	60.0	40.0	0.0	40.0	30.0	40.0	20.0	70.0	50.0	20.0
2 (3)	10.0	3.3	26.7	6.7	10.0	13.3	10.0	6.7	0.0	0.0	3.3	3.3	3.3	3.3
3 (3)	60.0	90.0	30.0	10.0	50.0	30.0	10.0	0.0	40.0	50.0	20.0	20.0	20.0	40.0
4 (3)	90.0	60.0	30.0	10.0	10.0	20.0	10.0	0.0	0.0	10.0	10.0	10.0	10.0	20.0
5 (4)	3.3	0.0	0.0	0.0	0.0	0.0	0.0	0.0	0.0	0.0	0.0	0.0	0.0	0.0
6 (4)	63.3	76.7	33.3	16.7	40.0	40.0	20.0	23.3	40.0	50.0	36.7	23.3	23.3	36.7
7 (4)	6.7	3.3	20.0	6.7	16.7	0.0	16.7	0.0	0.0	3.3	6.7	6.7	0.0	6.7
8 (4)	13.3	0.0	0.0	13.3	6.7	6.7	26.7	33.3	26.7	26.7	16.7	70.0	73.3	23.3
9 (4)	20.0	26.7	20.0	6.7	10.0	6.7	0.0	3.3	3.3	3.3	0.0	6.7	0.0	3.3
10 (4)	26.7	20.0	6.7	0.0	0.0	0.0	0.0	3.3	0.0	0.0	0.0	0.0	0.0	0.0
11 (4)	20.0	30.0	3.3	3.3	3.3	3.3	3.3	0.0	6.7	0.0	3.3	0.0	0.0	0.0
12 (4)	40.0	13.3	10.0	0.0	0.0	6.7	3.3	6.7	6.7	0.0	0.0	0.0	13.3	0.0
13 (5)	40.0	10.0	0.0	6.7	0.0	0.0	10.0	6.7	0.0	0.0	3.3	0.0	0.0	0.0
14 (5)	30.0	40.0	13.3	23.3	33.3	26.7	43.3	33.3	16.7	13.3	30.0	16.7	10.0	13.3
15 (5)	33.3	6.7	16.7	3.3	3.3	6.7	3.3	23.3	40.0	23.3	6.7	10.0	3.3	10.0
16 (5)	10.0	20.0	6.7	10.0	3.3	0.0	6.7	6.7	13.3	10.0	3.3	6.7	0.0	10.0
17 (5)	0.0	6.7	6.7	0.0	3.3	13.3	0.0	40.0	10.0	13.3	0.0	6.7	0.0	3.3
18 (5)	10.0	6.7	53.3	13.3	26.7	6.7	23.3	43.3	26.7	76.7	16.7	16.7	6.7	10.0
19 (5)	73.3	70.0	80.0	30.0	30.0	53.3	43.3	46.7	10.0	26.7	40.0	43.3	40.0	43.3
20 (5)	0.0	0.0	0.0	0.0	0.0	0.0	3.3	0.0	0.0	0.0	3.3	0.0	0.0	3.3
21 (1)	30.0	10.0	30.0	30.0	50.0	20.0	0.0	30.0	20.0	20.0				
22 (1)	70.0	0.0	30.0	10.0	10.0	10.0	0.0	50.0	10.0	10.0				
23 (1)	10.0	20.0	10.0	10.0	0.0	0.0	10.0	0.0	0.0	0.0				
24 (1)	0.0	0.0	20.0	0.0	20.0	10.0	0.0	0.0	0.0	20.0				
25 (6)	90.0	63.3	80.0	63.3	36.7	13.3	26.7							
26 (6)	86.7	36.7	13.3	20.0	3.3	3.3	3.3							
27 (6)	53.3	66.7	43.3	60.0	20.0	23.3	20.0							
28 (6)	40.0	10.0	6.7	16.7	30.0	13.3	10.0							
29 (6)	16.7	3.3	10.0	10.0	0.0	3.3	6.7							
30 (6)	13.3	16.7	6.7	6.7	3.3	0.0	6.7							
31 (6)	6.7	10.0	6.7	16.7	16.7	13.3	6.7							
32 (6)	3.3	6.7	6.7	6.7	3.3	6.7	10.0							

33 (6)	3.3	6.7	6.7	6.7	10.0	3.3	0.0							
34 (6)	0.0	3.3	3.3	0.0	0.0	0.0	0.0							
group	**NL-D1**													
rat	training day													
	**1**	**2**	**3**	**4**	**5**	**6**	**7**	**8**	**9**	**10**	**11**	**12**	**13**	**14**
35 (2)	**100.0**	**100.0**	**100.0**	**100.0**	50.0	40.0	10.0	10.0	30.0	10.0	10.0	**100.0**	0.0	10.0
36 (2)	**100.0**	**100.0**	**100.0**	50.0	90.0	**100.0**	60.0	60.0	20.0	**100.0**	40.0	40.0	40.0	70.0
37 (2)	**100.0**	**100.0**	**100.0**	**100.0**	**100.0**	60.0	30.0	0.0	10.0	30.0	20.0	50.0	40.0	30.0
38 (3)	**100.0**	**100.0**	**100.0**	**100.0**	**100.0**	30.0	40.0	40.0	20.0	30.0	40.0	50.0	30.0	30.0
39 (3)	**100.0**	**100.0**	**100.0**	**100.0**	40.0	20.0	20.0	30.0	30.0	20.0	70.0	50.0	30.0	20.0
40 (3)	**100.0**	**100.0**	**100.0**	80.0	30.0	30.0	50.0	40.0	60.0	50.0	40.0	20.0	40.0	60.0
41 (3)	**100.0**	50.0	40.0	**100.0**	20.0	50.0	20.0	60.0	60.0	20.0	30.0	70.0	20.0	20.0
42 (3)	**100.0**	**100.0**	20.0	20.0	20.0	50.0	**100.0**	**100.0**	**100.0**	**100.0**	**100.0**	**100.0**	60.0	50.0
43 (5)	**100.0**	**100.0**	80.0	96.7	**100.0**	**100.0**	93.3	33.3	20.0	76.7	**100.0**	23.3	36.7	**100.0**
44 (5)	**100.0**	**100.0**	**100.0**	90.0	**100.0**	66.7	**100.0**	**100.0**	**100.0**	90.0	73.3	76.7	50.0	43.3
45 (5)	**100.0**	**100.0**	**100.0**	**100.0**	**100.0**	83.3	56.7	66.7	93.3	50.0	56.7	56.7	26.7	26.7
46 (1)	**100.0**	80.0	**100.0**	50.0	**100.0**	40.0	20.0	40.0	10.0	30.0				
47 (6)	**100.0**	36.7	16.7	16.7	76.7	**100.0**	93.3							
48 (6)	**100.0**	20.0	13.3	6.7	10.0	0.0	10.0							
49 (6)	**100.0**	66.7	13.3	30.0	13.3	6.7	13.3							
50 (6)	**100.0**	**100.0**	**100.0**	**100.0**	**100.0**	66.7	36.7							
51 (6)	**100.0**	**100.0**	**100.0**	**100.0**	**100.0**	**100.0**	76.7							

group	**NL-SGL**													
rat	training day													
	**1**	**2**	**3**	**4**	**5**	**6**	**7**	**8**	**9**	**10**	**11**	**12**	**13**	**14**
52 (2)	60.0	30.0	70.0	30.0	60.0	70.0	40.0	10.0	**100.0**	30.0	40.0	60.0	60.0	50.0
53 (3)	80.0	**100.0**	70.0	70.0	40.0	40.0	0.0	30.0	10.0	0.0	10.0	10.0	10.0	10.0
54 (5)	56.7	66.7	**100.0**	90.0	23.3	26.7	26.7	43.3	53.3	33.3	63.3	43.3	36.7	46.7
55 (5)	36.7	6.7	3.3	13.3	6.7	10.0	30.0	30.0	46.7	60.0	**100.0**	53.3	26.7	50.0
56 (1)	40.0	50.0	60.0	20.0	20.0	30.0	60.0	**100.0**	10.0	60.0				
group	**NL-CEN**													
rat	training day													
	**1**	**2**	**3**	**4**	**5**	**6**	**7**	**8**	**9**	**10**	**11**	**12**	**13**	**14**
57 (2)	70.0	20.0	**100.0**	**100.0**	80.0	**100.0**	50.0	**100.0**	**100.0**	**100.0**	**100.0**	**100.0**	70.0	50.0
58 (3)	50.0	50.0	80.0	**100.0**	**100.0**	50.0	**100.0**	20.0	50.0	30.0	50.0	20.0	10.0	10.0
59 (5)	46.7	66.7	63.3	30.0	16.7	26.7	83.3	**100.0**	93.3	**100.0**	**100.0**	**100.0**	86.7	56.7
60 (5)	43.3	10.0	13.3	10.0	10.0	**100.0**	**100.0**	**100.0**	**100.0**	66.7	76.7	23.3	43.3	36.7
61 (5)	50.0	10.0	**100.0**	**100.0**	**100.0**	26.7	20.0	73.3	73.3	80.0	33.3	43.3	30.0	30.0
