# Supplementary material for: Rats that learn to vocalize for food reward emit longer and louder appetitive calls and fewer short aversive calls
Source: PLoS One. 2024 Feb 9;19(2):e0297174. doi: 10.1371/journal.pone.0297174 (PMC10857575; doi:10.1371/journal.pone.0297174)
Supplement: S2 Table — (PDF) [file pone.0297174.s005.pdf]

S2 Table. Percentage of rewards obtained and duration of training sessions in rats trained with several protocols of instrumental learning with USV emissions or nosepokes as rewarded responses; **a.** changes in percent of number of rewards obtained by rats in each USV training protocol, **b.** changes in the percent number of rewards and session duration in rats analyzed together; **c.** changes in session duration in nosepoke training; **d.** changes in number of nosepokes in test sessions; **e.** differences in percent number of rewards and session duration for the same rat (protocol 6) in USV vs. nosepoke training; the percentage of rewards achieved in the nosepoke-training was significantly higher than that obtained by the same rats in the previous USV-training; the duration of the first training session in both experiments was similar (p = 0.0688), while subsequent session duration became significantly shorter in nosepoke-training; see **S1 Fig**.

a

Protocol/Experiment	Friedman	Wilcoxon (first vs. last)
protocol 1 (A)	0.4402	0.9453
protocol 2 (B)	0.0515	0.3281
protocol 3 (C)	**<0.0001**	**0.0020**
protocol 4 (D)	0.3811	0.6523
protocol 5 (E)	**0.0470**	0.3092
protocol 6 (Skinner box, F)	0.3815	0.1875
protocol 6 (homecage, G)	**0.0143**	**0.0078**

b

Days/groups analyzed	Friedman		Wilcoxon (first vs. last)	
	% rewards (H)	session time (I)	% rewards (H)	session time (I)
days 1-7, only 7 trainings	**0.0089**	0.7292	**0.0015**	0.2324
days 1-7, all rats	**<0.0001**	0.8711	**0.0007**	0.9485
days 1-10, only 10 trainings	0.4402	0.4746	0.9453	0.7500
days 1-10, all rats	**0.0027**	0.9636	0.0858	0.1982
days 1-14, only 14 trainings	**<0.0001**	0.9648	**0.0022**	0.5412

c

Maximum number of rewards (K)	Friedman days 1-5	Wilcoxon day 1 vs. 5
10	**<0.0001**	**0.0078**
30	**<0.0001**	**<0.0001**

d

Maximum number of rewards (L)	Friedman days 1-3	Wilcoxon day 1 vs. 2	Wilcoxon day 1 vs. 3	Wilcoxon day 2 vs. 3
10	**0.0099**	**0.0078**	**0.0391**	0.4375
30	**<0.0001**	**<0.0001**	**<0.0001**	**0.0007**

e

Training day	Wilcoxon	
	% rewards	session time
1	**0.0020**	**0.0141**
2	**0.0002**	**<0.0001**
3	**<0.0001**	**<0.0001**
4	**<0.0001**	**<0.0001**
5	**0.0002**	**<0.0001**
