# Supplementary material for: Rats that learn to vocalize for food reward emit longer and louder appetitive calls and fewer short aversive calls
Source: PLoS One. 2024 Feb 9;19(2):e0297174. doi: 10.1371/journal.pone.0297174 (PMC10857575; doi:10.1371/journal.pone.0297174)
Supplement: S3 Table — (PDF) [file pone.0297174.s006.pdf]

S3 Table. Call rate (USV/min) in PL and NL rats; **a.** changes in call rate in PL and NL subgroups; **b.** differences in call rate between subgroups and PL-SUM and NL-SUM rats in training and test sessions; N/A – not applicable; **see Fig 3G and 3H**.

a

Test	Days analyzed	PL groups		NL groups				SUM groups	
	Training (GH)	MAX	PROG	CEN	D1	SGL	0	PL	NL
Friedman	days 1-7, all rats	0.4627	0.2806	0.6491	**0.0004**	0.2995	**<0.0001**	0.1426	**<0.0001**
Wilcoxon	day 1 vs. 7, all rats	0.6250	**0.0098**	>0.9999	**0.0039**	0.5000	**<0.0001**	0.1645	**<0.0001**
Friedman	days 1-14	0.5654	0.3847	0.4777	**0.0224**	0.5409	**0.0001**	N/A	N/A
Wilcoxon	day 1 vs. 14	N/A	0.0625	0.2500	0.2500	N/A	**0.0078**	N/A	N/A
	Test (GH)								
Friedman	days 1-3	0.1821	**0.0212**	0.3673	0.1333	0.6914	0.2602	**0.0288**	**0.0135**
Wilcoxon	day 1 vs. 3	0.3125	0.3750	0.1875	**0.0240**	>0.9999	0.0604	0.9341	**0.0012**

b

Training day (G)	Mann-Whitney							
	PL-MAX vs. PL-PROG	PL-MAX vs. NL-CEN	PL-MAX vs. NL-D1	PL-MAX vs. NL-SGL	PL-MAX vs. NL-0	PL-PROG vs. NL-CEN	PL-PROG vs. NL-D1	PL-PROG vs. NL-SGL
1	**0.0077**	**0.0357**	0.7972	**0.0357**	**<0.0001**	>0.9999	**0.0030**	0.6678
2	**0.0127**	**0.0159**	**0.0420**	**0.0357**	**<0.0001**	0.0759	0.6198	0.3007
3	**0.0400**	**0.0079**	**0.0120**	**0.0357**	**<0.0001**	0.9530	0.7802	0.2867
4	0.1275	**0.0079**	**0.0070**	**0.0357**	**<0.0001**	0.9530	0.8418	0.4685
5	0.1292	**0.0159**	**0.0420**	**0.0357**	**<0.0001**	0.3197	0.9682	0.3706
6	0.2544	**0.0357**	**0.0010**	**0.0357**	**<0.0001**	0.2168	0.3154	0.1608
7	0.1292	**0.0357**	**0.0010**	**0.0357**	**<0.0001**	0.2867	**0.0350**	**0.0140**
8	0.5333	0.8000	0.2667	0.2000	**0.0220**	0.9212	0.1535	**0.0485**
9	0.4222	0.4000	0.1333	0.2000	**0.0110**	0.2788	**0.0283**	**0.0485**
10	0.8889	0.4000	0.1333	0.2000	**0.0220**	0.2788	0.1535	**0.0485**
11	0.4286	0.8000	0.2000	0.3333	**0.0444**	0.9048	0.5476	0.6429
12	>0.9999	0.8000	0.2000	0.3333	**0.0444**	0.1667	**0.0238**	0.0714
13	0.8571	0.2000	0.2000	0.3333	**0.0444**	**0.0476**	**0.0476**	0.1429
14	0.4286	0.2000	0.8000	0.3333	**0.0444**	**0.0238**	0.0952	0.0714
Test day (G)								
1	0.3553	**0.0079**	**0.0013**	**0.0079**	**<0.0001**	0.0586	**0.0069**	**0.0373**
2	0.2544	**0.0317**	**0.0014**	**0.0159**	**<0.0001**	0.5135	**0.0209**	0.0753
3	0.2544	**0.0317**	**0.0021**	**0.0079**	**<0.0001**	0.9530	0.1743	0.1645
Training day (G)	PL-PROG vs. NL-0	NL-CEN vs. NL-D1	NL-CEN vs. NL-SGL	NL-CEN vs. NL-0	NL-D1 vs. NL-SGL	NL-D1 vs. NL-0	NL-SGL vs. NL-0	**PL-SUM vs. NL-SUM** (H)
1	0.0750	**0.0091**	0.7000	0.1483	**0.0091**	**<0.0001**	0.4413	0.0890
2	**0.0270**	**0.0196**	0.8571	>0.9999	0.1045	**0.0047**	0.6500	**0.0017**
3	**0.0241**	0.4376	0.5714	**0.0325**	0.1455	**0.0014**	0.9191	**0.0030**
4	0.0896	>0.9999	0.3929	**0.0289**	0.3636	**0.0151**	0.7057	**0.0062**
5	**0.0064**	0.1986	0.7143	0.2185	0.1455	**0.0027**	0.6226	**0.0006**
6	**0.0007**	0.7591	0.4000	0.1257	0.3727	**0.0055**	0.4887	**<0.0001**
7	**<0.0001**	0.8636	0.4000	**0.0300**	0.4818	**0.0024**	0.1548	**<0.0001**
8	**<0.0001**	0.4000	0.4000	**0.0176**	0.8571	0.0582	0.1011	**0.0002**
9	**<0.0001**	0.7143	0.7000	**0.0066**	0.8571	0.0604	0.1297	**<0.0001**
10	**0.0001**	>0.9999	0.4000	**0.0484**	0.6286	0.1104	0.1736	**<0.0001**
11	**0.0027**	>0.9999	0.8000	**0.0242**	0.9000	**0.0242**	**0.0444**	**0.0084**
12	**0.0007**	0.7000	0.9000	**0.0424**	0.8000	**0.0121**	**0.0444**	**<0.0001**
13	**0.0013**	0.4000	>0.9999	**0.0485**	0.8000	**0.0485**	0.2667	**<0.0001**
14	**0.0007**	0.1000	0.4000	0.2788	0.8000	**0.0121**	0.1778	**<0.0001**
Test day (G)								Test day (H)
1	**<0.0001**	0.5554	0.4206	0.0611	>0.9999	**0.0131**	0.2612	**<0.0001**
2	**0.0017**	0.4103	0.4206	**0.0394**	0.4109	0.2151	0.7058	**<0.0001**
3	**0.0002**	0.1364	0.0952	**0.0037**	0.6074	**0.0041**	0.1362	**<0.0001**
