# Supplementary material for: Rats that learn to vocalize for food reward emit longer and louder appetitive calls and fewer short aversive calls
Source: PLoS One. 2024 Feb 9;19(2):e0297174. doi: 10.1371/journal.pone.0297174 (PMC10857575; doi:10.1371/journal.pone.0297174)
Supplement: S4 Table — (PDF) [file pone.0297174.s007.pdf]

S4 Table. Session duration in NL and PL rats; **a.** changes in session duration in NL-SUM and PL-SUM rats; **b.** changes in session duration in PL-MAX and PL-PROG subgroups; **c.** differences in training sesson duration between PL-SUM and NL-SUM rats; N/A – not applicable; ND – no data; **see Fig 3I and 3J**.

a

Days analyzed (J)	NL-SUM			PL-SUM		
	Number of animals	Friedman	Wilcoxon, first vs. last	Number of animals	Friedman	Wilcoxon, first vs. last
days 1-7 (only 7 trainings)	n = 15	0.2267	0.0625	n = 5	**0.0154**	**0.0078**
days 1-7 (all rats)	n = 61	**0.0043**	**0.0053**	n = 15	**<0.0001**	**0.0002**
days 1-10 (only 10 trainings)	n = 6	0.6114	>0.9999	n = 2	N/A	ND
days 1-10 (all rats)	n = 46	**0.0275**	0.2412	n = 10	**<0.0001**	**0.0078**
days 1-14 (only 14 trainings)	n = 40	**0.0007**	**0.0029**	n = 8	**0.0002**	**0.0078**

b

Group analyzed (I)	Friedman	Wilcoxon (first vs. last)
PL-MAX, days 1-7, n = 5	**0.0011**	0.0625
PL-PROG, days 1-7, n = 10	**0.0003**	**0.0078**
PL-PROG, first 3 days with max number of rewards, n = 10	0.0665	**0.0195**

c

Training day (J)	Mann-Whitney
	PL-SUM vs. NL-SUM
1	0.1330
2	**0.0007**
3	**0.0014**
4	**0.0002**
5	**<0.0001**
6	**<0.0001**
7	**<0.0001**
8	**<0.0001**
9	**<0.0001**
10	**<0.0001**
11	**0.0001**
12	**<0.0001**
13	**<0.0001**
14	**<0.0001**
