# Supplementary material for: Rats that learn to vocalize for food reward emit longer and louder appetitive calls and fewer short aversive calls
Source: PLoS One. 2024 Feb 9;19(2):e0297174. doi: 10.1371/journal.pone.0297174 (PMC10857575; doi:10.1371/journal.pone.0297174)
Supplement: S5 Table — (PDF) [file pone.0297174.s008.pdf]

S5 Table. Percentage of rewarded USV in the total number of USV and total number of 50-kHz USV in rats during 7 and 14 training sessions; **a.** changes in percent of rewarded USV in rats with seven training sessions; **b.** changes in percent of rewarded USV in rats with fourteen training sessions; **c.** differences in percent of rewarded USV between subgroups of rats with 7 training sessions; **d.** differences in percent of rewarded USV between subgroups of rats with 14 training sessions; **see Fig 4**.

a

Group analyzed (AC)	Rats with 7 training sessions			
	Friedman		Wilcoxon (first vs. last)	
	rewarded USV / all USV	rewarded USV / 50-kHz USV	rewarded USV / all USV	rewarded USV / 50-kHz USV
PL-SUM	**0.0255**	**0.0059**	0.0625	0.0625
NL-SUM	0.4868	0.2727	0.4143	**0.0295**
NL-SUM/0	0.8989	0.0583	0.3125	0.0625
NL-0	0.2782	0.3004	0.9453	0.3594

b

Group analyzed (BD)	Rats with 14 training sessions			
	Friedman		Wilcoxon (first vs. last)	
	rewarded USV / all USV	rewarded USV / 50-kHz USV	rewarded USV / all USV	rewarded USV / 50-kHz USV
PL-SUM	**<0.0001**	**<0.0001**	**0.0313**	**0.0313**
NL-SUM	**<0.0001**	**<0.0001**	**0.0034**	**0.0034**
NL-SUM/0	**<0.0001**	**<0.0001**	**0.0156**	**0.0078**
NL-0	0.0748	**0.0032**	0.1484	0.2500

c

Training day	Rats with 7 training sessions, Mann-Whitney							
	rewarded USV / all USV (A)				rewarded USV / 50-kHz USV (C)			
	PL-SUM vs. NL-SUM	PL-SUM vs. NL-SUM/0	PL-SUM vs. NL-0	NL-SUM/0 vs. NL-0	PL-SUM vs. NL-SUM	PL-SUM vs. NL-SUM/0	PL-SUM vs. NL-0	NL-SUM/0 vs. NL-0
1	>0.9999	0.6905	0.8591	0.5941	0.5531	0.6905	0.5941	0.5941
2	0.9185	0.8413	0.7679	0.9794	0.3139	0.8413	0.2155	0.3210
3	0.0526	0.3095	**0.0400**	0.3097	0.5958	0.8413	0.5741	0.8844
4	0.2294	>0.9999	0.0932	0.2051	0.6598	0.8413	0.4396	0.3863
5	**0.0036**	0.2222	**0.0007**	**0.0123**	0.2570	0.5476	**0.0370**	0.0706
6	**0.0106**	0.0952	**0.0123**	0.3696	0.0842	0.3333	0.0719	0.6111
7	0.0845	0.5476	**0.0440**	0.2431	0.8775	0.8413	0.9710	0.5395

d

Training day	Rats with 14 training sessions, Mann-Whitney							
	rewarded USV / all USV (B)				rewarded USV / 50-kHz USV (D)			
	PL-SUM vs. NL-SUM	PL-SUM vs. NL-SUM/0	PL-SUM vs. NL-0	NL-SUM/0 vs. NL-0	PL-SUM vs. NL-SUM	PL-SUM vs. NL-SUM/0	PL-SUM vs. NL-0	NL-SUM/0 vs. NL-0
1	0.9016	0.7546	0.5468	0.1866	>0.9999	0.6620	0.6337	0.3147
2	0.6407	0.6620	0.1812	**0.0207**	0.7900	0.2684	0.5728	**0.0135**
3	0.9563	0.2824	0.2171	**0.0224**	0.3982	**0.0293**	0.5468	**0.0482**
4	0.3977	0.5728	**0.0290**	**0.0003**	0.9237	0.2284	0.2471	**0.0140**
5	0.0570	0.7546	**0.0013**	**0.0103**	0.2051	0.8518	**0.0047**	**0.0059**
6	0.0794	0.7296	**0.0043**	**0.0160**	0.3318	0.8518	**0.0338**	**0.0137**
7	0.1144	0.8518	**0.0073**	**0.0068**	0.4494	0.8518	0.1079	**0.0207**
8	0.2611	0.8518	0.0813	0.1049	0.7781	0.9217	0.5338	0.3203
9	0.2868	0.9497	0.0779	**0.0482**	0.9560	0.7546	0.6282	0.1994
10	0.0795	0.4136	**0.0283**	0.0688	0.3199	0.9497	0.1029	0.0878
11	**0.0060**	0.0813	**0.0023**	**0.0202**	**0.0274**	0.0463	0.0793	0.5543
12	**0.0017**	**0.0293**	**0.0013**	0.1540	**0.0033**	**0.0426**	**0.0023**	0.1893
13	**0.0002**	**0.0047**	**0.0007**	**0.0053**	**0.0004**	**0.0127**	**0.0007**	**0.0020**
14	**0.0034**	**0.0426**	**0.0027**	0.1949	**0.0313**	0.1006	**0.0260**	0.1689
