# Supplementary material for: Rats that learn to vocalize for food reward emit longer and louder appetitive calls and fewer short aversive calls
Source: PLoS One. 2024 Feb 9;19(2):e0297174. doi: 10.1371/journal.pone.0297174 (PMC10857575; doi:10.1371/journal.pone.0297174)
Supplement: S6 Table — (PDF) [file pone.0297174.s009.pdf]

S6 Table. Rewarded USV emitted in series: all rewarded USV emitted in series (number of USV), maximum number of USV in single series (max USV); **a.** changes in number of USV and maximum number of USV in PL-SUM and NL-SUM/0 rats; **b.** differences in the number of USV and maximum number of USV between PL-SUM and NL-SUM/0 rats; **c.** changes in USV duration in PL-SUM and NL-SUM/0 rats; **d.** differences in USV duration between PL-SUM and NL-SUM/0 rats.

a

Data analyzed	Group analyzed	Friedman		Wilcoxon (first vs. last)	
		7 trainings	14 trainings	7 trainings	14 trainings
Number of USV	PL-SUM	**0.0430**	**0.0010**	0.0625	0.0625
	NL-SUM/0	0.9303	**0.0153**	0.6875	0.1250
Max USV	PL-SUM	0.0551	**<0.0001**	0.0625	**0.0313**
	NL-SUM/0	0.6358	**0.0366**	>0.9999	**0.0469**

b

Training day	Mann-Whitney			
	Number of USV		Max USV	
	7 trainings	14 trainings	7 trainings	14 trainings
1	0.7143	0.7702	0.5635	0.5641
2	0.1190	0.2118	0.4603	0.3643
3	0.5794	0.7792	0.3175	0.7196
4	0.3889	0.8801	0.3571	0.4119
5	0.2063	0.8735	0.3175	0.4872
6	**0.0476**	0.1322	**0.0079**	0.3646
7	0.0873	0.0992	0.3810	0.7509
8		0.5724		0.8265
9		0.6427		0.8248
10		0.0749		0.1848
11		0.0629		**0.0077**
12		**0.0010**		0.2374
13		**0.0017**		**0.0090**
14		**0.0057**		0.0559

c

Group analyzed	Friedman		Wilcoxon (first vs. last)	
	7 trainings	14 trainings	7 trainings	14 trainings
PL-SUM	0.3509	0.4845	0.8125	0.3125
NL-SUM/0	0.0816	**0.0386**	0.0625	0.0781

d

Training day	Mann-Whitney	
	7 trainings	14 trainings
1	>0.9999	0.6065
2	0.8081	0.2398
3	0.9399	0.3277
4	0.9048	0.2673
5	0.9048	**0.0173**
6	**0.0357**	**0.0460**
7	**0.0159**	**0.0216**
8		0.2824
9		**0.0338**
10		**0.0027**
11		**0.0130**
12		**0.0010**
13		0.0813
14		**0.0013**
