# Supplementary material for: Rats that learn to vocalize for food reward emit longer and louder appetitive calls and fewer short aversive calls
Source: PLoS One. 2024 Feb 9;19(2):e0297174. doi: 10.1371/journal.pone.0297174 (PMC10857575; doi:10.1371/journal.pone.0297174)
Supplement: S7 Table — (PDF) [file pone.0297174.s010.pdf]

**S7 Table. Duration of 50-kHz USV**; **a.** changes in 50-kHz USV duration in habituation and training session in rats with 7 or 14 training sessions or when pooled together; **b.** changes in 50-kHz USV duration in test sessions in rats with 7 or 14 training sessions; **c.** differences in 50-kHz USV duration between PL and NL groups in rats with seven training sessions; **d.** differences in 50-kHz USV duration between PL and NL groups in rats with fourteen training sessions; **see Fig 5**.

**a**

| Group analyzed | Friedman | | | | Wilcoxon (first vs. last) | | | |
|---|---|---|---|---|---|---|---|---|
| | Number of trainings | | | | | | | |
| | 4* + 7 (A) | 7 (A) | 14 (C) | 7 (all rats) | 4* + 7 (A) | 7 (A) | 14 (C) | 7 (all rats) |
| PL-SUM | **0.0050** | **0.0442** | 0.5608 | **0.0092** | 0.1250 | 0.1250 | 0.4375 | **0.0205** |
| NL-SUM | 0.0759 | 0.1073 | 0.3158 | 0.3079 | **0.0215** | 0.0554 | 0.2979 | 0.2185 |
| NL-SUM/0 | 0.0683 | **0.0479** | 0.5557 | 0.0683 | 0.0625 | 0.0625 | 0.1484 | **0.0327** |
| NL-0 | 0.6452 | 0.7226 | 0.5808 | 0.9168 | 0.2324 | 0.4922 | 0.9453 | 0.7019 |

* – including 4 habituations.

**b**

| Group analyzed | Friedman | | Wilcoxon (first vs. last) | |
|---|---|---|---|---|
| | 7 trainings, test (B) | 14 trainings, test (D) | 7 trainings, test (B) | 14 trainings, test (D) |
| PL-SUM | 0.9537 | 0.4419 | >0.9999 | 0.4375 |
| NL-SUM | 0.8187 | **0.0104** | 0.7729 | **0.0036** |
| NL-SUM/0 | 0.3637 | 0.0789 | 0.5000 | **0.0391** |
| NL-0 | 0.9737 | 0.1197 | 0.8457 | 0.0781 |

**c**

| Days analyzed (AB) | Mann-Whitney | | | |
|---|---|---|---|---|
| | PL-SUM vs. NL-SUM | PL-SUM vs. NL-SUM/0 | PL-SUM vs. NL-0 | NL-SUM/0 vs. NL-0 |
| habituation 1 | 0.6240 | 0.8413 | 0.3863 | 0.0753 |
| habituation 2 | 0.2234 | 0.6905 | 0.1645 | 0.7000 |
| habituation 3 | 0.4545 | 0.6905 | 0.1742 | 0.3710 |
| habituation 4 | **0.0146** | 0.2222 | **0.0080** | 0.3097 |
| training 1 | 0.3398 | 0.5000 | 0.0753 | **0.0077** |
| training 2 | **0.0048** | 0.2222 | **0.0010** | **0.0223** |
| training 3 | 0.0633 | 0.4206 | **0.0400** | 0.5331 |
| training 4 | 0.1916 | 0.9683 | 0.0703 | 0.2065 |
| training 5 | **0.0051** | 0.2222 | **0.0013** | 0.3863 |
| training 6 | **<0.0001** | **0.0079** | **0.0007** | 0.6167 |
| training 7 | **0.0023** | **0.0079** | **0.0113** | 0.9291 |
| test 1 | **0.0193** | 0.1508 | **0.0193** | 0.8591 |
| test 2 | 0.1613 | 0.3095 | 0.1941 | 0.8362 |
| test 3 | 0.0951 | 0.4206 | 0.0706 | 0.1965 |

**d**

| Days analyzed (CD) | Mann-Whitney | | | |
|---|---|---|---|---|
| | PL-SUM vs. NL-SUM | PL-SUM vs. NL-SUM/0 | PL-SUM vs. NL-0 | NL-SUM/0 vs. NL-0 |
| training 1 | 0.0980 | 0.6620 | **0.0127** | **0.0379** |
| training 2 | 0.0677 | 0.3450 | **0.0266** | **0.0134** |
| training 3 | **0.0016** | **0.0117** | **0.0043** | 0.4418 |
| training 4 | **0.0256** | 0.0813 | **0.0386** | 0.0608 |
| training 5 | **0.0008** | **0.0087** | **0.0027** | 0.1605 |
| training 6 | **0.0018** | **0.0216** | **0.0027** | **0.0379** |
| training 7 | **0.0008** | **0.0093** | **0.0027** | 0.1605 |
| training 8 | **0.0133** | 0.0593 | **0.0200** | 0.1605 |
| training 9 | **0.0008** | **0.0077** | **0.0027** | 0.5054 |
| training 10 | **0.0005** | **0.0013** | **0.0080** | 0.1049 |
| training 11 | **0.0005** | **0.0013** | **0.0080** | **0.0281** |
| training 12 | **0.0032** | **0.0080** | **0.0200** | 0.5941 |
| training 13 | **<0.0001** | **0.0007** | **0.0007** | **0.0115** |
| training 14 | **0.0002** | **0.0027** | **0.0013** | 0.3671 |
| test 1 | **<0.0001** | **0.0013** | **0.0005** | 0.3154 |
| test 2 | **0.0006** | **0.0047** | **0.0027** | 0.1654 |
| test 3 | **0.0002** | **0.0077** | **0.0002** | **0.0023** |
