# Supplementary material for: Rats that learn to vocalize for food reward emit longer and louder appetitive calls and fewer short aversive calls
Source: PLoS One. 2024 Feb 9;19(2):e0297174. doi: 10.1371/journal.pone.0297174 (PMC10857575; doi:10.1371/journal.pone.0297174)
Supplement: S8 Table — (PDF) [file pone.0297174.s011.pdf]

S8 Table. Amplitude of 50-kHz USV; **a.** changes in 50-kHz USV amplitude in habituation and training session in rats with 7 or 14 training sessions or when pooled together; **b.** changes in 50-kHz USV amplitude in test sessions in rats with 7 or 14 training sessions; **c.** differences in 50-kHz USV amplitude between PL-SUM and NL-SUM/0 groups in rats with 7 training sessions; **d.** differences in 50-kHz USV amplitude between PL-SUM and NL-SUM/0 groups in rats with 14 training sessions; **see Fig 5**.

a

Group analyzed	Friedman				Wilcoxon (first vs. last)			
	Number of trainings							
	4* + 7 (A)	7 (A)	14 (C)	7 (all rats)	4* + 7 (A)	7 (A)	14 (C)	7 (all rats)
PL-SUM	**0.0038**	**0.0224**	0.9475	0.0538	0.1875	0.1250	0.3125	0.0674
NL-SUM/0	**0.0217**	0.0745	0.4679	0.5766	0.6250	0.3125	0.2500	0.4548

* – including 4 habituations.

b

Group analyzed	Friedman		Wilcoxon (first vs. last)	
	7 trainings, test (B)	14 trainings, test (D)	7 trainings, test (B)	14 trainings, test (D)
PL-SUM	0.1821	0.1416	0.4375	0.0938
NL-SUM/0	0.9537	0.9674	0.8125	0.9453

c

Days analyzed (EF)	Mann-Whitney
	PL-SUM vs. NL-SUM/0
habituation 1	0.3095
habituation 2	0.6905
habituation 3	0.5476
habituation 4	0.6905
training 1	0.8413
training 2	0.3095
training 3	0.5476
training 4	>0.9999
training 5	0.2222
training 6	0.4206
training 7	0.3095
test 1	0.2222
test 2	0.2222
test 3	0.3095

d

Days analyzed (GH)	Mann-Whitney
	PL-SUM vs. NL-SUM/0
training 1	0.4908
training 2	0.3450
training 3	0.2284
training 4	0.2824
training 5	0.3450
training 6	0.0813
training 7	0.1812
training 8	0.1079
training 9	0.1812
training 10	0.0593
training 11	0.1419
training 12	**0.0200**
training 13	**0.0007**
training 14	**0.0080**
test 1	**0.0426**
test 2	0.4908
test 3	0.1419
