# Supplementary material for: Rats that learn to vocalize for food reward emit longer and louder appetitive calls and fewer short aversive calls
Source: PLoS One. 2024 Feb 9;19(2):e0297174. doi: 10.1371/journal.pone.0297174 (PMC10857575; doi:10.1371/journal.pone.0297174)
Supplement: S9 Table — (PDF) [file pone.0297174.s012.pdf]

**S9 Table. Percent of short 22-kHz USV in all USV**; **a.** changes in percent of short 22-kHz USV in PL and NL groups, rats with 7 or 14 training days; **b.** differences in percent of short 22-kHz USV between PL and NL rats with 7 training days; **c.** differences in percent of short 22-kHz USV between PL and NL rats with 14 training days; **d.** changes in percent of short 22-kHz in PL/NL groups in test sessions; **e.** differences in percent of short 22-kHz USV between PL and NL groups in test sessions, rats with 7 or 14 training days; **see Fig 6**.

**a**

| Group analyzed | Friedman | | | Wilcoxon (first vs. last) | | |
|---|---|---|---|---|---|---|
| | 4 habituations +7 trainings (A) | 7 trainings (A) | 14 trainings (C) | 4 habituations +7 trainings (A) | 7 trainings (A) | 14 trainings (C) |
| PL-SUM | 0.9092 | 0.9190 | **0.0197** | >0.9999 | 0.8125 | 0.0625 |
| NL-SUM | **<0.0001** | **0.0015** | 0.2674 | **0.0006** | **0.0302** | 0.4637 |
| NL-SUM/0 | **0.0218** | **0.0311** | 0.5130 | 0.1250 | 0.2324 | 0.7422 |
| NL-0 | **<0.0001** | 0.0627 | 0.2390 | **0.0098** | 0.0625 | 0.4609 |

**b**

| Day analyzed (A) | Mann-Whitney | | | |
|---|---|---|---|---|
| | PL-SUM vs. NL-SUM | PL-SUM vs. NL-SUM/0 | PL-SUM vs. NL-0 | NL-SUM/0 vs. NL-0 |
| habituation 1 | 0.8658 | 0.3095 | 0.7429 | 0.2041 |
| habituation 2 | 0.9286 | 0.8413 | >0.9999 | 0.7599 |
| habituation 3 | 0.6532 | 0.2857 | 0.2041 | 0.1672 |
| habituation 4 | 0.6848 | 0.8333 | 0.6950 | 0.9817 |
| training 1 | 0.0544 | 0.8968 | **0.0027** | **0.0027** |
| training 2 | **0.0325** | 0.4603 | **0.0083** | 0.0786 |
| training 3 | **0.0035** | 0.1508 | **0.0013** | 0.1225 |
| training 4 | **0.0109** | 0.2698 | **0.0030** | 0.1365 |
| training 5 | **0.0026** | 0.1587 | **0.0007** | 0.0949 |
| training 6 | **0.0005** | **0.0317** | **0.0007** | 0.1645 |
| training 7 | **0.0051** | 0.0556 | **0.0077** | 0.1961 |

**c**

| Training day (C) | Mann-Whitney | | | |
|---|---|---|---|---|
| | PL-SUM vs. NL-SUM | PL-SUM vs. NL-SUM/0 | PL-SUM vs. NL-0 | NL-SUM/0 vs. NL-0 |
| 1 | 0.1978 | 0.7546 | 0.0593 | 0.0830 |
| 2 | **0.0100** | 0.0813 | **0.0070** | **0.0353** |
| 3 | **0.0319** | 0.2151 | **0.0127** | 0.0799 |
| 4 | **0.0414** | 0.2394 | **0.0176** | **0.0075** |
| 5 | **0.0024** | **0.0193** | **0.0047** | 0.1949 |
| 6 | **0.0103** | **0.0283** | **0.0290** | 0.1304 |
| 7 | **0.0045** | 0.1066 | **0.0007** | 0.1540 |
| 8 | **0.0022** | 0.0533 | **0.0007** | 0.1372 |
| 9 | **0.0030** | 0.0703 | **0.0007** | 0.0779 |
| 10 | **0.0017** | **0.0290** | **0.0013** | 0.6454 |
| 11 | **0.0018** | **0.0310** | **0.0013** | **0.0070** |
| 12 | **0.0005** | **0.0113** | **0.0007** | 0.3663 |
| 13 | **0.0002** | **0.0027** | **0.0013** | 0.0650 |
| 14 | **<0.0001** | **0.0013** | **0.0007** | 0.6671 |

**d**

| Group analyzed | 7 trainings (B) | | 14 trainings (D) | |
|---|---|---|---|---|
| | Friedman, days 1-3 | Wilcoxon, day 1 vs. 3 | Friedman, days 1-3 | Wilcoxon, day 1 vs. 3 |
| PL-SUM | 0.3673 | 0.3125 | 0.2014 | 0.1250 |
| NL-SUM | 0.6271 | 0.7197 | 0.4323 | 0.6685 |
| NL-SUM/0 | 0.9537 | 0.8125 | 0.6543 | 0.3125 |
| NL-0 | 0.3675 | 0.7695 | 0.1853 | 0.1953 |

**e**

| Test day | Mann-Whitney | | | |
|---|---|---|---|---|
| 7 trainings (B) | PL-SUM vs. NL-SUM | PL-SUM vs. NL-0 | PL-SUM vs. NL-SUM/0 | NL-SUM/0 vs. NL-0 |
| 1 | 0.5500 | 0.5674 | 0.6905 | 0.8561 |
| 2 | 0.1147 | 0.0949 | 0.4206 | 0.4216 |
| 3 | 0.4550 | 0.2065 | 0.7460 | 0.2544 |
| 14 trainings (D) | | | | |
| 1 | **0.0257** | **0.0127** | 0.1615 | 0.0822 |
| 2 | **0.0075** | **0.0130** | **0.0303** | 0.2233 |
| 3 | **0.0485** | 0.1795 | **0.0426** | 0.9329 |
